# Cell-free tumor DNA analysis in advanced or metastatic breast cancer patients: mutation frequencies, testing intention, and clinical impact

**DOI:** 10.1093/pcmedi/pbae034

**Published:** 2024-12-24

**Authors:** Hanna Huebner, Pauline Wimberger, Elena Laakmann, Eugen Ruckhäberle, Matthias Ruebner, Sarah Lehle, Sabrina Uhrig, Philipp Ziegler, Theresa Link, Carolin C Hack, Erik Belleville, Iris Faull, Marcus Hausch, Diethelm Wallwiener, Andreas Schneeweiss, Hans Tesch, Sara Y Brucker, Matthias W Beckmann, Peter A Fasching, Volkmar Müller, Tanja N Fehm

**Affiliations:** Department of Gynecology and Obstetrics, Erlangen University Hospital, Comprehensive Cancer Center Erlangen-EMN, Friedrich Alexander University of Erlangen–Nuremberg, Erlangen 91054, Germany; Bavarian Cancer Research Center (BZKF), Erlangen 91054, Germany; Department of Gynecology and Obstetrics, Carl Gustav Carus Faculty of Medicine and University Hospital, Dresden, TU 01307, Germany; National Center for Tumor Diseases (NCT), Dresden 01307, Germany; German Cancer Research Center (DKFZ), Heidelberg 69120, Germany; Carl Gustav Carus Faculty of Medicine and University Hospital, Dresden, TU 01307, Germany; Helmholtz-Zentrum Dresden-Rossendorf (HZDR), Dresden 01307, Germany; German Cancer Consortium (DKTK), Dresden 01307, Germany; German Cancer Research Center (DKFZ), Heidelberg 69120, Germany; Department of Gynecology, Hamburg-Eppendorf University Medical Center, Hamburg 20246, Germany; Department of Gynecology and Obstetrics, CIO ABCD, University Hospital Düsseldorf, Düsseldorf 40225, Germany; Department of Gynecology and Obstetrics, Erlangen University Hospital, Comprehensive Cancer Center Erlangen-EMN, Friedrich Alexander University of Erlangen–Nuremberg, Erlangen 91054, Germany; Bavarian Cancer Research Center (BZKF), Erlangen 91054, Germany; Department of Gynecology and Obstetrics, Erlangen University Hospital, Comprehensive Cancer Center Erlangen-EMN, Friedrich Alexander University of Erlangen–Nuremberg, Erlangen 91054, Germany; Bavarian Cancer Research Center (BZKF), Erlangen 91054, Germany; Department of Gynecology and Obstetrics, Erlangen University Hospital, Comprehensive Cancer Center Erlangen-EMN, Friedrich Alexander University of Erlangen–Nuremberg, Erlangen 91054, Germany; Bavarian Cancer Research Center (BZKF), Erlangen 91054, Germany; Department of Gynecology and Obstetrics, Erlangen University Hospital, Comprehensive Cancer Center Erlangen-EMN, Friedrich Alexander University of Erlangen–Nuremberg, Erlangen 91054, Germany; Bavarian Cancer Research Center (BZKF), Erlangen 91054, Germany; Department of Gynecology and Obstetrics, Carl Gustav Carus Faculty of Medicine and University Hospital, Dresden, TU 01307, Germany; National Center for Tumor Diseases (NCT), Dresden 01307, Germany; German Cancer Research Center (DKFZ), Heidelberg 69120, Germany; Carl Gustav Carus Faculty of Medicine and University Hospital, Dresden, TU 01307, Germany; Helmholtz-Zentrum Dresden-Rossendorf (HZDR), Dresden 01307, Germany; German Cancer Consortium (DKTK), Dresden 01307, Germany; German Cancer Research Center (DKFZ), Heidelberg 69120, Germany; Department of Gynecology and Obstetrics, Erlangen University Hospital, Comprehensive Cancer Center Erlangen-EMN, Friedrich Alexander University of Erlangen–Nuremberg, Erlangen 91054, Germany; Bavarian Cancer Research Center (BZKF), Erlangen 91054, Germany; ClinSol GmbH & Co. KG, Würzburg 97074, Germany; Guardant Health, Inc., Redwood City, CA 94063, USA; Guardant Health, Inc., Redwood City, CA 94063, USA; Department of Obstetrics and Gynecology, University of Tübingen, Tübingen 72076, Germany; National Center for Tumor Diseases, Heidelberg University Hospital, German Cancer Research Center (DKFZ), Heidelberg 69120, Germany; Oncology Practice at Bethanien Hospital Frankfurt, Frankfurt am Main 60389, Germany; Department of Obstetrics and Gynecology, University of Tübingen, Tübingen 72076, Germany; Department of Gynecology and Obstetrics, Erlangen University Hospital, Comprehensive Cancer Center Erlangen-EMN, Friedrich Alexander University of Erlangen–Nuremberg, Erlangen 91054, Germany; Bavarian Cancer Research Center (BZKF), Erlangen 91054, Germany; Department of Gynecology and Obstetrics, Erlangen University Hospital, Comprehensive Cancer Center Erlangen-EMN, Friedrich Alexander University of Erlangen–Nuremberg, Erlangen 91054, Germany; Bavarian Cancer Research Center (BZKF), Erlangen 91054, Germany; Department of Gynecology, Hamburg-Eppendorf University Medical Center, Hamburg 20246, Germany; Department of Gynecology and Obstetrics, CIO ABCD, University Hospital Düsseldorf, Düsseldorf 40225, Germany

**Keywords:** ctDNA, cfDNA, cell-free DNA, breast cancer, genetic testing, Clinical trial registration No.: NCT02338167

## Abstract

**Background:**

Circulating cell-free tumor DNA (ctDNA) provides a non-invasive approach for assessing somatic alterations. The German PRAEGNANT registry study aims to explore molecular biomarkers and investigate their integration into clinical practice. In this context, ctDNA testing was included to understand the motivations of clinicians to initiate testing, to identify somatic alterations, and to assess the clinical impact of the results obtained.

**Methods:**

Patients with advanced/metastatic breast cancer were prospectively enrolled in the Prospective Academic Translational Research Network for the Optimization of Oncological Health Care Quality in the Adjuvant and Advanced/Metastatic Setting (PRAEGNANT study; NCT02338167). The FDA-approved and CE-marked GUARDANT360 CDx test was used to assess somatic alterations. A ctDNA-analysis report was provided to the treating physician along with a questionnaire about the intent for testing and the clinical implications of test results.

**Results:**

ctDNA from 49 patients was analyzed prospectively: 37 (76%) had at least one somatic alteration in the analyzed geneset; 14 patients (29%) harbored alterations in *TP53*, 12 (24%) in *PIK3CA*, and 6 (12%) in *ESR1*. Somatic mutations in *BRCA1* or *BRCA2* were detected in 3 (6%) and 4 (8%) patients, respectively, and 59% of patients had hormone receptor-positive, human epidermal growth factor receptor 2-negative breast cancer. Questionnaires regarding test intentions and clinical impact were completed for 48 (98%) patients. These showed that ctDNA testing influenced treatment decisions for 35% of patients.

**Discussion:**

The high prevalence of somatic alterations in *TP53, PIK3CA, ESR1*, and *BRCA1/2* genes, identified by ctDNA genotyping, highlights their potential as biomarkers for targeted therapies. Detection of specific mutations affected treatment decisions, such as eligibility for alpelisib, and might further facilitate treatment with e.g. elacestrant or capiversatib in future treatment lines.

## Introduction

### Genomic profiling of breast cancer

Genetic alterations are a key feature to determine the best treatment approach for patients with breast cancer and other solid tumors. Alterations in germline DNA are well described to be associated with treatment outcome and, for example, analysis of germline *BRCA1* or *BRCA2* mutations has been established as a companion diagnostic for the poly ADP ribose polymerase inhibitor (PARPi) olaparib [1]. Within the last couple of years, however, somatic alterations have also become increasingly important for precision medicine. Specifically, for patients with hormone receptor-positive (HR+) breast cancer subtypes the treatment landscape based on tumor mutation profiles has significantly evolved. HR+ tumors are known to undergo significant genomic profile changes from the primary tumor herd through metastatic progression [2], and somatic alterations are commonly acquired during endocrine therapy and contribute to treatment resistance [3]. Common alterations involve mutations in the *estrogen receptor 1* (*ESR1*) region [4], mutations in the mitogen-activated protein (MAP) kinase pathway, and alterations in various transcription factors [5], such as *ARID1A* [6]. Recent approvals for somatic alterations as companion diagnostics underscore the importance of testing. Food and Drug Administration (FDA) approvals include alpelisib for postmenopausal women and men with HR+, human epidermal growth factor receptor 2-negative (HER2-) advanced or metastatic, *PIK3CA*-mutated breast cancer, elacestrant for postmenopausal women or adult men with estrogen receptor positive (ER+), HER2-, advanced or metastatic, *ESR1*-mutated breast cancer, and capivasertib for patients with HR+, HER2-, locally advanced or metastatic breast cancer harboring one or more *PIK3CA, AKT1*, or *PTEN* alterations [7–9]. Companion diagnostic tests such as the therascreen PIK3CA RGQ PCR Kit for *PIK3CA* mutation analysis and the Guardant360 CDx assay for *ESR1* testing in plasma samples have also received FDA approval in this context.

Somatic DNA alterations can be assessed either by analysis of the tumor material itself [e.g. formalin-fixed paraffin-embedded samples (FFPE)] taken by an invasive biopsy or during surgery or by analysis of circulating tumor DNA (ctDNA) in the plasma of cancer patients, obtained through a peripheral blood draw [10–12]. In contrast to samples taken by biopsy or surgery, the analysis of ctDNA is minimally invasive and allows for continuous monitoring, evaluation of spatial and temporal heterogeneity, and representation of even minimal tumor burden. The importance of analyzing and monitoring ctDNA was demonstrated within the PAlbociclib and Circulating Tumor DNA for ESR1 Mutation Detection (PADA-1) trial. The PADA-1 study, involving 1017 women with advanced HR+ and HER2- breast cancer showed that switching to fulvestrant upon mutation detection doubled the duration of progression-free survival (PFS) compared to standard therapy [8]. In addition, several ongoing and completed trials are exploring the use of somatic alterations to guide treatment decisions. The plasmaMATCH trial, focusing on ctDNA analysis in advanced breast cancer, and the COGNITION-Guide and the CATCH trials in Germany, utilizing whole-genome/exome and transcriptome sequencing, aim to tailor therapies based on individual genetic profiles [13, 14]. The SOLAR-1 trial, the INAVO120 trial as well as the EMERALD and PADA trials are outstanding examples of the potential of ctDNA testing for patient and therapy selection [7, 8, 15]. Further phase III studies are currently ongoing focusing on e.g. selective estrogen receptor degraders and their combination with CDK4/6 inhibitors or other endocrine therapeutic drugs [16].

### Feasibility of ctDNA testing in Germany

Given the evolving therapeutic landscape, characterized by the introduction of elacestrant, alpelisib, and capivasertib, and the upcoming inavolisib, molecular companion diagnostics will become an integral part of treatment decision-making. The current status of mutation testing, specifically in ctDNA, within Germany and other EU countries, however, is still not yet completely established. With the approval of elacestrant by the European Commission in September 2023, the first treatment for patients with breast cancer with a necessity for ctDNA testing became part of routine setting. In this context, *ESR1* mutation testing in ctDNA is recommended by the European Society for Medical Oncology (ESMO) Precision Medicine Working Group [17]. Furthermore, while there is the option of solely testing the *ESR1* gene region, there are multiple testing approaches [targeted or next-generation sequencing (NGS)] that enable the analysis of a wider gene panel—as recommended by the ESMO NGS recommendation 2024. Such panel testings, however, also enable the detection of alterations in genes for non-approved treatment approaches or treatments for other cancer entities. In this context, the consequences of such genetic test results and the clinical impact for the patients still need to be evaluated. The testing of certain biomarkers within the German registry study Prospective Academic Translational Research Network for the Optimization of Oncological Health Care Quality in the Adjuvant and Advanced/Metastatic Setting (PRAEGNANT) was shown to be a promising approach not only to enable the provision of research-based test results to patients but also for enrollment of patients into clinical trials [18, 19]. In this context, the presented project aimed to provide a validated panel-testing of ctDNA to patients enrolled in the PRAEGNANT registry study and to evaluate the intention of testing by the treating physician and the clinical impact of test results.

## Materials and methods

### Patients

Patients were enrolled in the prospective registry study PRAEGNANT (ClinicalTrials.gov identifier: NCT02338167) [20], which is an ongoing, prospective, multicentric breast cancer registry in hospitals and practices across Germany. The main focus of the PRAEGNANT registry is to provide real-world data on the treatment landscape [21], conduct biomarker research [22], and enable the return of individual research results [19]. Patients with advanced or metastatic breast cancer are eligible for inclusion at any time point of their disease. Patient and disease data were collected at study entry and follow-up visits. All patients provided informed consent at enrollment. The study was approved by the respective German ethics committees (ethical approval number: 234/2014BO1, first approval on 17 June 2014, approval of Amendment 1 on 11 June 2015, approval of Amendment 2 on 18 March 2019; Ethics Committee of the Medical Faculty, University of Tübingen, Tübingen, Germany). A subproject (PRAEGNANT 360°) of the PRAEGNANT study was conducted between February 2022 and August 2023 at four different study sites within Germany. The PRAEGNANT 360° subproject included ctDNA testing for any patient enrolled in the main study based on physician's choice. No other inclusion or exclusion criteria were defined for the subproject.

### ctDNA testing

Whole blood samples were collected using Streck cell-free DNA (cfDNA) BCT tubes at the respective study site and sent to the PRAEGNANT central biobank unit. Shipment to the PRAEGNANT central biobank unit was obligatory due to study protocol requirements. In routine care, however, samples can be shipped directly at room temperature from the point of blood collection to Guardant Health's laboratory. The PRAEGNANT laboratory initiated the shipment of the Streck tubes, kept at room temperature, to the GUARDANT Health central laboratory in Redwood City, CA, USA, for cfDNA extraction and analysis of ctDNA. Guardant Health has a proprietary tagging system that allows barcoding of the ctDNA fragments out of the total cfDNA, increasing the sequence efficiency by 2-3 times compared with other technologies. NGS analysis was performed at the GUARDANT Health central laboratory. The FDA-approved and Council of the European Union (CE)-marked GUARDANT360 CDx test, a 74-gene panel including all guideline-recommended biomarkers, including single nucleotide variants (SNVs), indels, fusions, amplifications, and microsatellite instability, was employed. Blood samples were analyzed and a GUARDANT360 CDx report was generated and published through the secure GUARDANT Health portal.

ctDNA analysis often results in secondary germline findings. According to established standards, mutation calls with variant allele frequencies (VAF) of 40%–60% (heterozygous) or 95%–100% (homozygous) are likely single nucleotide polymorphisms present in germline DNA [23, 24]. Within the study collective, only one alteration was observed with a VAF between 60% and 95% (SMAD4 D351N, VAF: 66%). This specific alteration is likely of germline origin, according to ClinVar documentation. Therefore, we applied a cut-off of <40% VAF to define somatic alterations. In order to define patients with ctDNA-positive disease a cutoff of ≥0.4% VAF was set in accordance with recent publications [25, 26]. This cutoff was used solely to determine the number of patients with ctDNA positivity.

### Return of research results

The GUARDANT360 CDx report was merged with the original PRAEGNANT patient ID at the central biobank unit, printed, and returned to the relevant study site and respective treating physician. Each report was accompanied by (i) a questionnaire and (ii) a letter to the treating physician. The letter stated that, due to the pseudonymization of patient and biomaterial data, the correct assignment of test results could not be guaranteed and that validation of test results within the clinical routine is recommended.

### Questionnaire

Each treating physician who received the GUARDANT360 CDx report was asked to complete a questionnaire about the intention of testing and the clinical impact of test results. The questionnaire included 11 questions in the German language, referring to the reason for testing and whether the results influenced any treatment decisions taken. All questions were either single-choice questions or open questions for free-text answers. Completed questionnaires were returned to the central biobank unit of the PRAEGNANT registry study for analysis.

### Statistical considerations

Results are presented in numbers and percentages. Data were analyzed using IBM SPSS statistics Version 24. Graphs were created using GraphPad Prism Version 9.5.1 and Microsoft Excel Version 2108.

## Results

### Test initiation

**Figure 1.**
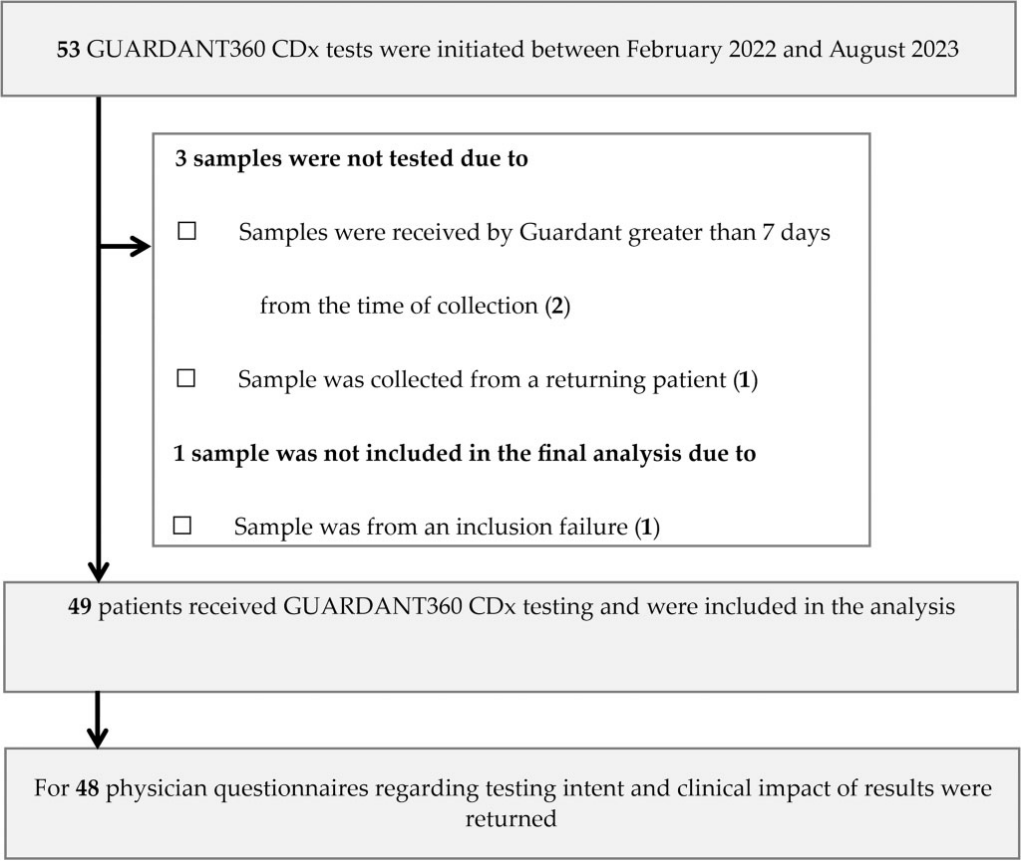
Patient flow chart.

A total of 53 tests for 52 patients were initiated by physicians and shipped to the PRAEGNANT central biobank. The median shipment time from blood draw to arrival at the GUARDANT Health central laboratory was 4 days (range: 1–18 days). Sample stability in the GUARDANT Streck tubes has been validated for up to 7 days to ensure safe transport to the laboratory. Usually, samples from Europe arrive at the lab in 24–72 hours. Of those 53 samples, 3 were not eligible for testing due to prolonged shipment time (>7 days, *n* = 2) to the GUARDANT Health central laboratory or due to being from a returning patient (*n* = 1). One patient was excluded from the final analysis due to inclusion failure (Fig. 1). All samples (*n* = 49, 100%) that were received by the GUARDANT Health central laboratory within 7 days after blood draw were successfully sequenced. Thus, ctDNA from 49 patients was tested and included in the analysis. The mean age of patients included in the study was 48.4 years at primary diagnosis and 55.5 years at time of ctDNA testing (Table 1). Most patients included were HR+ and HER2- (*n* = 29, 59%, Table 1). In all, 18 patients (51%) of the full study group were in the 1st line of therapy at time of test initiation and 12 (48%) belonged to the HR+/HER2- cohort (Table 1); 6 (17%) patients had an HER2-amplified and 5 (14%) patients a triple negative tumor.

**Table 1.** Patient characteristics.

Characteristic		All patients (*n* = 49)	HR+, HER2- (*n* = 29)
Age at primary diagnosis (years)	Mean (SD)	48.4 (10.5)	51.1 (10.7)
	Missing, *n*	7	1
Age at time of ctDNA test (years)	Mean (SD)	55.5 (12.1)	57.8 (10.8)
	Missing, *n*	2	1
Body mass index (kg/m^2^)	Mean (SD)	26.3 (4.3)	27.2 (3.6)
	Missing, *n*	26	13
Clinical tumor subtype	TNBC^a^, *n* (%)	5 (14)	0 (0)
	Luminal A-like, *n* (%)	14 (40)	14 (58)
	Luminal B-like, *n* (%)	10 (29)	10 (42)
	HER2-positive, *n* (%)	6 (17)	0 (0)
	Missing, *n*	14	5
Grading	1, *n* (%)	2 (60)	2 (8)
	2, *n* (%)	18 (50)	12 (50)
	3, *n* (%)	16 (44)	10 (42)
	Missing, *n*	13	0 (0)
Metastatic site	Brain, *n* (%)	5 (13)	2 (7)
	Visceral, *n* (%)	22 (55)	17 (61)
	Bone, *n* (%)	6 (15)	4 (14)
	Other, *n* (%)	7 (18)	5 (18)
	Missing, *n*	9	1
Therapy situation at time of ctDNA test	1st line, *n* (%)	18 (51%)	12 (48%)
	2nd line, *n* (%)	7 (20%)	5 (20%)
	3rd line and higher, *n* (%)	10 (29%)	8 (32%)
	Missing, *n*	14	4

a TNBC, triple negative breast cancer.

### Frequencies of genetic alterations

**Figure 2.**
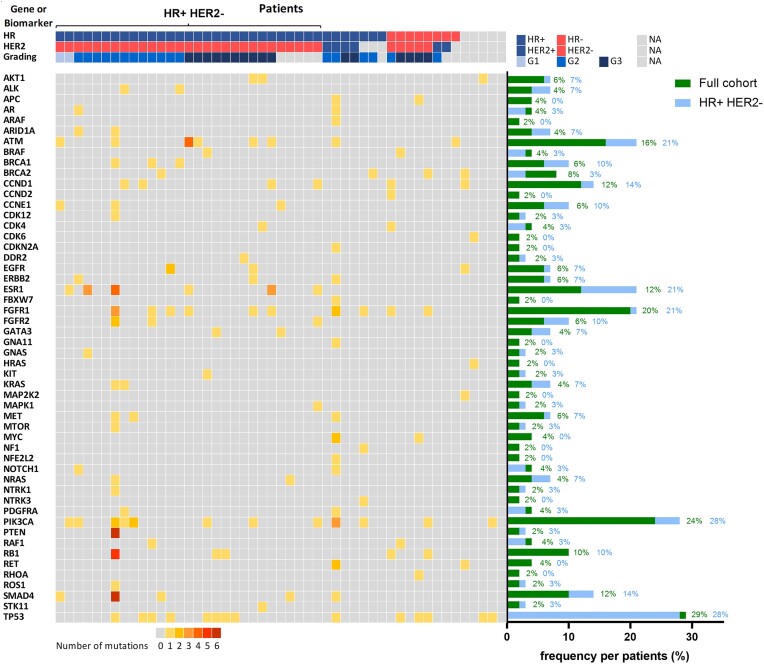
Frequencies and distributions of alterations in ctDNA. Heatmap of alterations detected in ctDNA of analyzed patients (*n* = 49). Number of alterations detected are marked by color coding. Patients are grouped by HR and HER2 expression and tumor grading. Frequencies of affected genes are presented for the full cohort (green, *n* = 49) and the HR+/HER2- cohort (blue, *n* = 29).

In total, 39 (80%) patients harbored at least one alteration in one of the analyzed genes and 33 (67%) in more than one gene. Of those alterations, 9 were detected with a variant allele fraction >40% and thus were marked as potentially germline mutations and excluded from the subsequent somatic mutation analysis. Based on this cut-off, 37 (76%) patients harbored at least one somatic alteration and 32 (65%) in more than one gene (Fig. 2). ctDNA positivity based on a cutoff of VAF ≥ 0.4% was detected for 31 (63%) patients. The most common genes affected were *TP53* (*n* = 14, 29%), *PIK3CA* (*n* = 12, 24%), *FGFR1* (*n* = 10, 20%), and *ATM* (*n* = 8, 16%) (Fig. 2). Somatic mutations in *BRCA1* or *BRCA2* were detected for 3 (6%) and 4 (8%) patients, respectively. One *BRCA2* mutation was categorized as synonymous and one *BRCA2* and three *BRCA1* alterations as variants of uncertain significance. For 6 patients (12%) an alteration in ESR1 was detected (Fig. 2).

In the subgroup of HR+/HER2- patients, 8 (28%) patients had an alteration in TP53, 8 (28%) in *PIK3CA*, 6 (21%) in *FGFR1*, and 6 (21%) in *ATM* (Fig. 2). Alterations in *BRCA1* and *BRCA2* were detected for 3 (10%) and 1 (3%) cases, respectively. All 6 patients with an *ESR1* alteration of the full cohort had HR+ and HER2- breast cancer, representing 21% of the HR+/HER2- subgroup (Fig. 2).

**Figure 3.**
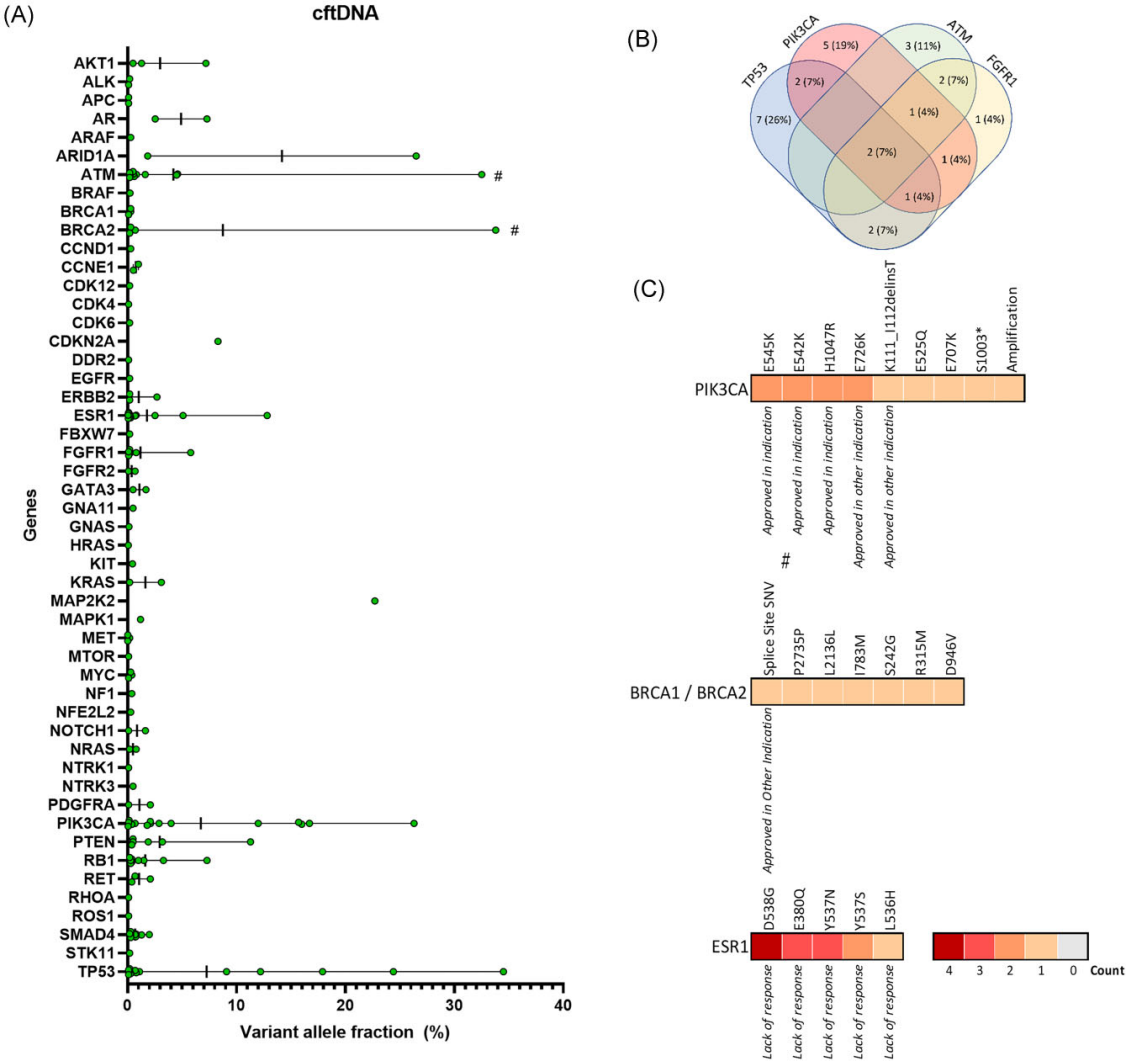
Variant allele fraction and shared mutations per patient. (**A**) VAF of alterations in analyzed genes are presented as mean ± range. Only alterations with a VAF < 40% (non-germline) are shown. Green dots indicate VAF of individual alterations detected within the listed genes. (**B**) Venn diagram with the four most frequent mutated genes including numbers (%) of patients with one or more of the listed genes affected. (**C**) Gene variant distribution for the targetable genes *PIK3CA, BRCA1/2*, and *ESR1*. Variant heat maps show the frequency of individual variants within the full cohort. The legend shows the numbers of patients with the respective variant detected in ctDNA. Variants associated with approved treatment options are marked by the approval indication. The hash (#) indicates alterations with a VAF ≤ 40% that were marked as suspicious for germline mutation in the GUARDANT Health report.

Somatic alterations (<40% VAF) were further analyzed in regards to the mean VAF per gene. The highest mean VAF was detected for alterations in *MAP2K2* (mean 22.70%, *n* = 1), *ARID1A* (mean 14.18%, range 1.85%–26.50%) and *BRCA2* (mean 8.76%, range 0.20%–33.80%, Fig. 3A). Alterations in *BRCA1* had a mean VAF of 0.23% (range 0.10%–0.30%) and those in *ESR1* of 1.75% (range 0.05%–12.81%, Fig. 3A). In all, 11 (22%) patients had alterations (deletion/mutation or amplification) in more than one out of the four most frequent affected genes (*TP53, PIK3CA, FGFR1, and ATM*; Fig. 3B). The most common somatic mutations in *PIK3CA* were E545K, E726K, E542K, and H1047R (*n* = 2, each) and in *ESR1* D538G (*n* = 4; Fig. 3C).

### Detected druggable mutations

The GUARDANT360 CDx report provides treatment suggestions [for European Medicines Agency (EMA) approved therapies] based on the detected alterations. Those treatment suggestions can either be approved for the cancer of interest (here, breast cancer) or for other indications. In addition, alterations associated with a lack of response (indicating resistance mechanisms) to certain treatment options are reported as well. Druggable somatic mutations approved for breast cancer were detected for 8 (16%), for other indications for 13 (27%) and lack of response for 7 (14%) patients (Table 2). The molecular consequences of those alterations were either a missense, nonsense, or frameshift or inframe insertions/deletions or splice variants, and thus affect protein structure (Table 2). Treatment options approved for other indications were identified based on alterations in the following genes: *ATM, ERBB2, PIK3CA, BRCA2, RET, NF1, EGFR*, and *MET*. These alterations led to the suggestion of various off-label or clinical trial treatments, including olaparib, trastuzumab deruxtecan, alpelisib, rucaparib, talazoparib, pralsetinib, selpercatinib, selumetinib, afatinib, dacomitinib, erlotinib, gefitinib, osimertinib, and tepotinib (Table 2). Lack of treatment response was reported for alterations detected in the *ESR1, FGFR2*, and *RET* gene loci. Lack of response based on an *ESR1* mutation was indicated for anastrozole, exemestane, and letrozole (Table 2).

**Table 2.** Druggable mutations.

Patient	Gene	Alteration^a^	Molecular consequence	VAF (%)	Treatment^b^	Indication^b^
2	PIK3CA	H1047R	Missense	2.13	Alpelisib + fulvestrant	Breast cancer
18	PIK3CA	E545K	Missense	0.4	Alpelisib	Breast cancer
27	PIK3CA	E545K	Missense	4.0	Alpelisib	Breast cancer
28	PIK3CA	E542K	Missense	0.06	Alpelisib	Breast cancer
30	PIK3CA	H1047R	Missense	12.0	Alpelisib	Breast cancer
36	PIK3CA	E545K	Missense	2.1	Alpelisib	Breast cancer
37	PIK3CA	H1047R	Missense	1.8	Alpelisib	Breast cancer
46	PIK3CA	E545K	Missense	16.7	Alpelisib	Breast cancer
46	PIK3CA	E542K	Missense	0.7	Alpelisib	Breast cancer
1	ATM	G956fs	Frameshift	0.23	Olaparib	Other indication
2	ERBB2	V777L	Missense	2.7	Trastuzumab deruxtecan	Other indication
7	PIK3CA	K111_I112delinsT	Inframe insertion/deletion	26.3	Alpelisib	Other indication
19	ERBB2	A775_G776insYVMA	Inframe insertion	0.2	Trastuzumab, trastuzumab deruxtecan	Other indication
19	ATM	R805*	Nonsense	4.6	Olaparib	Other indication
23	ATM	G2897fs	Frameshift	32.52	Olaparib	Other indication
28	PIK3CA	E726K	Missense	0.1	Alpelisib	Other indication
31	RET	V804M	Missense	2.1	Pralsetinib, selpercatinib	Other indication
37	NF1	Splice site SNV	Splice site variant	0.4	Selumetinib	Other indication
40	BRCA2	Splice site SNV	Splice site variant	33.8	Olaparib, rucaparib, talazoparib	Other indication
41	EGFR	T725M	Missense	0.2	Afatinib, dacomitinib, erlotinib, gefitinib, osimertinib	Other indication
46	MET	Exon 14 skipping SNV	Splice site variant	0.02	Tepotinib	Other indication
47	PIK3CA	E726K	Missense	16.0	Alpelisib	Other indication
48	ATM	E2039K	Missense	0.2	Olaparib	Other indication
1	ESR1	Y537S	Missense	2.53	Anastrozole, exemestane, letrozole	Lack of response
4	ESR1	Y537N	Missense	12.81	Anastrozole, exemestane, letrozole	Lack of response
4	ESR1	E380Q	Missense	0.25	Anastrozole, exemestane, letrozole	Lack of response
4	ESR1	D538G	Missense	0.05	Anastrozole, exemestane, letrozole	Lack of response
7	ESR1	E380Q	Missense	0.07	Anastrozole, exemestane, letrozole	Lack of response
7	ESR1	Y537N	Missense	0.08	Anastrozole, exemestane, letrozole	Lack of response
7	ESR1	Y537S	Missense	0.1	Anastrozole, exemestane, letrozole	Lack of response
7	ESR1	D538G	Missense	0.8	Anastrozole, exemestane, letrozole	Lack of response
7	FGFR2	N549K	Missense	0.7	Infigratinib	Lack of response
27	ESR1	D538G	Missense	0.7	Anastrozole, exemestane, letrozole	Lack of response
30	ESR1	E380Q	Missense	5.1	Anastrozole, exemestane, letrozole	Lack of response
30	FGFR2	N549K	Missense	0.09	Infigratinib	Lack of response
31	RET	V804M	Missense	2.1	Cabozantinib, vandetanib	Lack of response
44	ESR1	D538G	Missense	0.2	Anastrozole, exemestane, letrozole	Lack of response
44	ESR1	L536H	Missense	0.2	Anastrozole, exemestane, letrozole	Lack of response
44	ESR1	Y537N	Missense	0.3	Anastrozole, exemestane, letrozole	Lack of response

a Only alterations with a VAF < 40% are presented.

b As suggested by GUARDANT CDx report.

c For breast cancer treatment approved in case of a proven germline mutation.

### Intention of testing and clinical impact

Questionnaires regarding the intention of testing and clinical impact were completed by the treating physicians for 48 patients (98%). Based on the answers given, 29% of patients (*n* = 14) had an indication for PARPi treatment at the time of test initiation in the case of a detected *BRCA1* or *2* mutation and 19% (*n* = 9) for alpelisib in the case of a *PI3KCA* mutation (Table 3); 56% (*n* = 27) and 54% (*n* = 26) had an indication for treatment with a PARPi or alpelisib at the time of the next change of therapy in the case of a detected *BRCA1/2* or *PI3KCA* alteration (Table 3). For 3 patients (6%) neither an indication for PARPi or alpelisib therapy was reported. For 2 of those, the treating physician responded that an indication for another genomic alteration was present from the time of the next change of therapy (Table 3).

**Table 3.** Indication before initiation of ctDNA testing.

Question	Answer	*n* (%)
Did the patient have an indication for a PARP inhibitor if a *BRCA1/2* mutation had been detected during testing?	Yes, at present	14 (29.2%)
	Yes, from the next change of therapy	27 (56.2%)
	No	7 (14.6%)
	Total	48 (100.0%)
Did the patient have an indication for therapy with alpelisib if a *PI3KCA* mutation had been detected during testing?	Yes, at present	9 (18.7%)
	Yes, from the next change of therapy	26 (54.2%)
	No	13 (27.1%)
	Total	48 (100.0%)
If both questions were answered in the negative, did the patient have an indication for another genomic alteration-based indication for targeted therapy? ^a,b^	Yes, at present	0 (0.0%)
	Yes, from the next change of therapy	2 (66.7%)
	No	1 (33.3%)
	Total	3 (100.0%)

a Group: Did the patient have an indication for a PARP inhibitor in the case of a BRCA1/2Mutation = No.

b Group: Did the patient have an indication for therapy with alpelisib in the case of PI3KCA = No.

Overall, ctDNA testing influenced current or future treatment decisions for 35% of patients (*n* = 17) (Table 4). Of those, a treatment decision towards PARPi therapy was made for 12% (*n* = 2), towards PI3K inhibitor therapy for 41% (*n* = 7) and other therapeutic decisions for 74% of the patients, with the possibility of selecting multiple options accounting for the total exceeding 100% (Table 4). Treating physicians responded that patients had a benefit from GUARDANT360 CDx testing for 59% (*n* = 27) of the patients successfully tested. For most of patients (*n* = 34, 74%), the results from GUARDANT360 CDx testing were not validated on tumor tissue—even though validation was recommended in the accompanying result letter, due to the pseudonymization of data (Table 4). This remained the case at least until the completion of the questionnaire. Of those validated on FFPE tissue, the majority of responders indicated that the mutations identified by GUARDANT360 CDx testing were not detected in the FFPE tumor material (*n* = 7, 70%) (Table 4).

**Table 4.** Actions taken after ctDNA testing.

Question	Answer	*n* (%)
Did the results of Guardant testing influence current or future treatment decisions?	Yes	17 (35.4%)
	No	31 (64.6%)
	Total	48 (100.0%)
If yes, what treatment decision did you make based on the testing?^a^	PARP inhibitor therapy	2 (11.8%)
	PI3K inhibitor therapy	7 (41.2%)
	Other therapeutic decision	9 (52.9%)
	Total	18 (105.9%^e^)
Do you think the patient had a benefit from the results from the Guardant testing?	Yes	27 (58.7%)
	No	19 (41.3%)
	Total	46 (100.0%)
	Missing	2
Have the results from the Guardant testing been validated on tumor tissue?	Yes, all of them	0 (0.0%)
	Yes, but not all of them	12 (26.1%)
	No, none of them	34 (73.9%)
	Total	46 (100.0%)
	Missing	2
If only selected mutations were validated, which were they?^b^	PIK3CA	7 (58.33%)
	BRCAness-HRD/HRD	2 (16.67%)
	HRAS	1 (8.33%)
	BRCA1	1 (8.33%)
	FGFR2	1 (8.33%)
	Other	2 (16.67%)
	Total	14 (116.65%^e^)
	Missing	2
If the results were validated on the tumor tissue, which material was used for validation?^c^	Primary tumor (FFPE)	1 (9.1%)
	Metastasis (FFPE)	10 (90.9%)
	cfDNA/blood	0 (0.0%)
	Total	11 (100.0%)
	Missing	1
If the results were validated on the FFPE tumor, have the identified mutations also been detected in the validaton process?^d^	Yes, all of them	3 (30.0%)
	No	7 (70.0%)
	Total	10 (100.0%)
	Missing	1

a Group: Did the results of the Guardant test influence current or future treatment decisions? = Yes.

b Group: Have the results from the Guardant testing been validated on the tumor = Yes, but not all of them.

c Group: Have the results from the Guardant testing been validated on the tumor = Yes.

d Group: Which material was used for validation = FFPE (primary tumor or metastasis).

e The question was a multiple-choice question, therefore the percentage is >100%.

## Discussion

In summary, the PRAEGNANT 360° subproject revealed a ctDNA detection rate of 76% and a ctDNA-positivity (based on VAF ≥ 0.4% criterium) of 63%, with the most frequent alterations in *TP53, PIK3CA, FGFR1*, and *ATM*. The incidence was in line with results from similar trials. In a real-world cohort of patients with HR+/HER2- metastatic breast cancer, ctDNA was detected in 65% of cases [27]. A recent study evaluated ctDNA alterations in a cohort of metastatic patients with ER+/HER- disease. Of those, 96% had detectable ctDNA of which 69% had a ctDNA-positive disease defined by VAF ≥ 0.4% [28]. ctDNA detection rates vary based on sequencing methods used and the number of targets defined. A study analyzing ctDNA from 233 patients with metastatic breast cancer using target-capture deep sequencing with the focus on 1021 frequently mutated genes, found ctDNA alteration (VAF ≥ 1%) in 85% of cases [29]. Consistent with our results, *TP53* alterations were the most common, occurring in 36% of cases [29]. Additionally, the prevalence of *PIK3CA* mutations (26%) and *BRCA1/2* mutations (10%) aligned closely with our findings (24% and 14% within the PRAEGNANT full cohort) [29]. However, the incidence of *ESR1* alterations was meaningfully lower (6%) compared to our PRAEGNANT full cohort (12%) [29]. While the PRAEGNANT cohort was solely selected based on physician's discretion, the patients tested by Liu *et al*. [29] had triple negative or HR+/HER2+ metastatic breast cancer and, thus, had not received endocrine treatment. Endocrine therapy is known to promote clonal expansion of *ESR1* mutant tumor cells, leading to endocrine resistance [30]. Over 59% of the PRAEGNANT cohort comprised patients with HR+/HER2- cancer, likely all having undergone some form of endocrine treatment; 21% of patients in this subgroup tested positive for actionable *ESR1* mutations. A pooled analysis of multiple trials assessing *ESR1* mutations in hormone-sensitive breast cancer revealed an overall incidence of 23% (95% confidence interval 18%–28%), consistent with our findings [31]. Differences in incidences are mainly due to varying study populations, pre-treatments, and other selection criteria. Clinical trials including patients who have received prior aromatase inhibitor treatment showed *ESR1* mutation frequencies ranging from 26% to 44% [8, 32–34, 31, 35, 36]. Within our setting, elacestrant or other treatment options for *ESR1* mutated cancer cases were not yet approved in Germany. Thus, patients chosen for GUARDANT360 CDx testing were not selected due to *ESR1* testing options, probably resulting in a slightly different subpopulation cohort compared to other current trials focusing on endocrine resistance.

Genetic testing of ctDNA will become increasingly important for treatment decisions within routine cancer care. Findings from various studies show the impact of ctDNA analysis for tailoring targeted treatments. Current treatment options include elacestrant for *ESR1* mutated tumors, alpelisib for *PIK3CA* mutations, and PARPi for germline *BRCA1/2* alterations [8, 37, 38]. Additional genetic locations of interest are under investigation. For example, capivasertib demonstrated clinically meaningful activity in patients with an *PIK3CA, PTEN*, or *AKT* mutated metastatic breast cancer [9, 39]. A large study including 1 462 patients with HER2-negative metastatic breast cancer, showed that targeted therapies matched to genomic alterations can improve PFS [40]. Besides genetic testing in advanced or metastatic breast cancer cases, ctDNA testing also shows promise as a tool for monitoring early breast cancer. Persistence of ctDNA during neoadjuvant therapy has been associated with poor treatment response, while mutation tracking via ctDNA has enabled the identification of patients at high risk of relapse. Additionally, ctDNA clearance has been correlated with improved survival outcomes. This highlights the potential relevance of ctDNA testing in routine clinical practice, not only for advanced but also for early breast cancer treatment approaches [41–43].

In Germany and many other countries, the testing for mutations in genes like *ESR1* will likely be primarily conducted by the responsible pathologist using single-gene testing methods (e.g. PCR-based). This approach is driven by financial considerations and the requirements of insurance reimbursement. While testing one individual gene region of interest is probably the most cost-effective compared to panel- or whole genome sequencing, it has to be taken into account that patients might benefit from the detection of alterations which are not yet approved as a companion diagnostic. ESMO recommendations for the use of liquid biopsy include the testing for *ESR1, PIK3CA, BRCA1*/*2, MSI-H, ERBB2* amplification, and *NTRK 1/2/3* fusion. *ESR1* mutations are recommended to be tested in ctDNA, while *ERBB2* amplification and *NTRK* fusion should only be tested when an advanced tissue biopsy is not available [17]. Within the PRAEGNANT cohort, we could detect somatic alterations that were not approved for breast cancer but for other indications for 27% of the analyzed cases. These affected genes were *ATM, ERBB2, PIK3CA, BRCA2, RET, NF1, EGFR*, and *MET*. In Germany, the CATCH trial—a prospective precision oncology program that guides treatment decision by performing whole genome and RNA sequencing—is currently evaluating the impact of whole genome sequencing on treatment outcome. Within the first 200 patients enrolled, 53 were evaluable and of those 40% achieved a stable disease and 30% an improvement in PFS [44]. However, it is still under discussion whether sequencing of the tumor tissue or ctDNA results in an actual survival benefit. A recent retrospective observational study evaluated the usefulness and real-world outcomes of NGS testing in patients with cancer [45]. It was shown that sequencing did not result in a PFS benefit for patients with a rapidly progressing cancer, short expected lifetime, or no standard therapeutic options [45]. Similarly, the BRE12-158 phase II trial focusing on patients with triple negative breast cancer and residual disease after neoadjuvant chemotherapy showed that genomically directed therapy was not superior to treatment of physician choice after neoadjuvant chemotherapy [46]. Both disease-free and overall-survival were assessed. However, enrollment of patients was between 2014 and 2018. Since then, targeted therapy options have increased in number and thus current patients might have a different outcome. Targeted panel sequencing might be a good option to enable a broader analysis of somatic alterations while not spending too much money on whole genome sequencing without a clear benefit.

Within the PRAEGNANT 360° subproject, for 2 cases (4%) physicians made a treatment decision towards a PARPi therapy, for 7 (14%) towards a PI3K inhibitor therapy, and an additional 9 patients (18%) received a different, unspecified therapy due to the provided panel testing. This subproject provides insight into the real-world potential of ctDNA panel testing. It underscores the impact of such results for clinical guidance and treatment decision making Interestingly, out of 10 cases for which the ctDNA results were validated using FFPE tumor tissue, 7 (70%) did not show the same ctDNA alterations in FFPE tumor material. Concordance between FFPE and ctDNA differs between cancer types [47]. However, high concordance was reported for breast cancer ctDNA and FFPE probes [14, 48]. A study focusing on *ESR1* mutations in FFPE DNA and ctDNA showed a concordance rate of 91% [49]. Within our study, only 26% of the ctDNA results obtained were validated on tumor tissue. Thus, the 70% discordance represents only a small percentage of the total alterations. In addition, no information on the timing of FFPE tumor material and blood collection is available. Treatment changes might have occurred in between, resulting in new occurrence of genetic alterations like *ESR1* mutations. In addition, the decision to validate certain results might be biased by e.g. low VAFs, unexpected alterations, or other unspecified factors. For future studies it would be of interest to further evaluate the clinical consequences taken if a validation was not successful. The lack of direct comparison of somatic alterations detected in ctDNA versus those in tumor biopsies is a limitation of our study. The discordance between FFPE and ctDNA testing was based on the physician's response to a questionnaire. Thus, no data on the type of surgery, the site of surgery, or the timing of sample collection is available. The project was designed solely based on ctDNA analysis due to not all patients having a tumor biopsy available at the time of testing and blood-based analysis being less invasive and thus more convenient. However, as previous analysis within the plasmaMATCH trial, a multicentre, multicohort, phase 2a, platform trial, demonstrated high agreement between ctDNA digital PCR and targeted sequencing (GUARDANT test) of 96%–99%, and a sensitivity of 93% overall and 98% with contemporaneous biopsies, validation of ctDNA test results is no longer needed [10, 14]. The sensitivity of targeted sequencing was shown to be at a high level of 90.9% [10] and respective tests are currently FDA- and CE-approved as companion diagnostics, which underlines their importance for clinical decision-making. Other previous studies also showed similar results of high concordance rates between contemporary tissue NGS sequencing analysis and Guardant360 Cdx results, which was mandatory for FDA approval [26].

The discrepancy between FFPE and ctDNA test results reported by the treating physicians within this subproject of the PRAEGNANT study could be attributed to several factors: tumor heterogeneity, timing of sample collection, selection bias in validation, and technical sensitivity differences. Tumor heterogeneity and sample timing may explain discrepancies between ctDNA and FFPE results. While FFPE tissue reflects a single tumor region, ctDNA testing captures DNA shed from multiple tumor sites, potentially providing a more comprehensive mutation profile [50, 51]. Additionally, ctDNA offers a “real-time” snapshot of tumor genetics, whereas FFPE samples often represent an earlier state, missing mutations that emerge over time [52]. Often, the primary tumor from surgery dissection is used for testing, which is not ideal for metastatic cancer cases. Selection bias in choosing specific ctDNA mutations for FFPE validation and differences in the technical sensitivity of each method may further contribute to discordance, as ctDNA tests can detect low-frequency mutations that FFPE might miss.

## Conclusions

The study identified key genetic mutations using ctDNA testing, serving as markers for targeted therapies. It underscores the clinical impact of such test results and highlights the importance of implementing ctDNA test strategies in the routine setting. In light of the recent approval of elacestrant for the treatment of *ESR1*-mutated tumors and the associated ctDNA companion diagnostic, as well as other future targeted treatment options, panel testing for ctDNA analyses should be strongly considered for implementation into clinical care in Germany. Further, the findings support considering patients with identified genetic mutations from routine panel testing as potential candidates for enrollment into clinical trials where ctDNA mutations serve as inclusion criteria for treatment.

## Data Availability

The datasets generated during and/or analyzed during the current study are not publicly available due to GDPR regulations but are available from the corresponding author on reasonable request.
